# Numerical simulation of pneumatic throttle check valve using computational fluid dynamics (CFD)

**DOI:** 10.1038/s41598-023-29457-4

**Published:** 2023-02-11

**Authors:** Marta Żyłka, Natalia Marszałek, Wojciech Żyłka

**Affiliations:** 1 The Faculty of Mechanical Engineering and Aeronautics, Department of Aerospace Engineering, Rzeszow University of Technology, av. Powstańców Warszawy 8, 35-959 Rzeszów, Poland; 2 Institute of Materials Engineering, College of Natural Sciences, University of Rzeszow, Pigonia 1, 35-310 Rzeszów, Poland

**Keywords:** Engineering, Mechanical engineering, Computational science

## Abstract

The article presents a numerical CFD simulation of a throttle-check valve used in an innovative control system for two pneumatic drives. This type of control is used in an innovative rehabilitation device for lower limbs. In order to determine the boundary conditions, experimental tests were carried out. The throttle valves on the test stand were scaled and the air flow rate values were read for different valve opening heights. The purpose of this article is to present a CFD simulation of a pre-adjusted check valve throttle. Numerical simulation (CFD) makes it possible to study the flow phenomena inside a pneumatic throttle-check valve, with different sizes of flow gaps. The obtained results made it possible to determine the distribution of physical quantities of static pressure, the velocity of the medium flowing through the valve, or the vector velocity distribution. The throttle valve assembly has been scaled for a suitable degree of synchronization of the movement of the piston actuators independently of the different external loads acting on each of them. The authors investigated airflow phenomena for different valve opening heights. The simulation provided information on the occurrence of supersonic and subsonic flow velocities at specific valve opening heights.

## Introduction

In systems where pneumatic drives are used, simultaneous movement of the piston rods is necessary. When there is a different external load, uneven movement of the drive piston rods is observed. It is difficult to obtain the same displacement of drive piston rods under different load^1,2^, mainly due to the fact that compressed air is compressible^3–5^, and there is also movement resistance caused by self-friction of drive pistons^6–10^.

Pneumatic systems use proportional valves^11^ and on/off solenoid valves^12,13^, with which to regulate air flow^5,14^. On/off solenoid valves are commonly used in industry because they are pneumatic components that are less expensive than proportional valves^15,16^.

To obtain the simultaneous movement of pneumatic drives, for example, motion synchronizer^17,18^ throttle or throttle-check valves^7,19^ are used. Throttle check valves are widely used in pneumatics as flow control elements in many industries.

The most common pneumatic elements used to regulate the flow of the working medium are throttle valves and throttle-check valves. However, the disadvantage of this valve is the sensitivity to changes in the load force of the piston rod drive. The flow of the working medium through the valve gap increases with the load force^20^.

The throttle-check valve is used to regulate the speed of the extension or retraction of the piston rods of pneumatic cylinders. It is a parallel connection of a throttle and a non-return valve. In this valve, the air flow is throttled in one direction only. The air flows through an adjustable reduced cross-section in the throttle valve, and the flow of the working medium closes the check valve. When moving in the opposite direction, the air flows freely with the open non-return valve^7^.

In hydraulic systems, motion synchronizers are used. The synchronization movement of hydraulic drives is usually carried out by a proportional valve or servo valve^21^. You can also meet with high-speed on–off valves (HSVs)^22^. HSV is used, for example, for pressure control^23^, and also for position control^24^. The authors of the paper^25,26^ presented the control of hydraulic drive elements through a high-speed on–off valve. The authors used a cooperative synchronization control algorithm, PWM–PFM (pulse-width modulation–pulse frequency modulation). In the literature^27,28^, the authors designed a controller to implement control with tracking of the velocity trajectory of the drives' piston rods.

The throttling valves investigated in this paper are key elements of the control of the rehabilitation device, which should be fully predictable and reliable due to its future use by patients after severe injuries, including spine. Therefore, simulations of their work were carried out to fully understand their operation. Knowledge of CFD simulation tests, throttle valve (differently scaled), became necessary in order to fully analyze the operation of the device and complete the planned design of the motion synchronizer system. The design knowledge of this type of pneumatic components is strictly protected by the manufacturers and is not available.

## Drive speed control system

**Figure 1:**
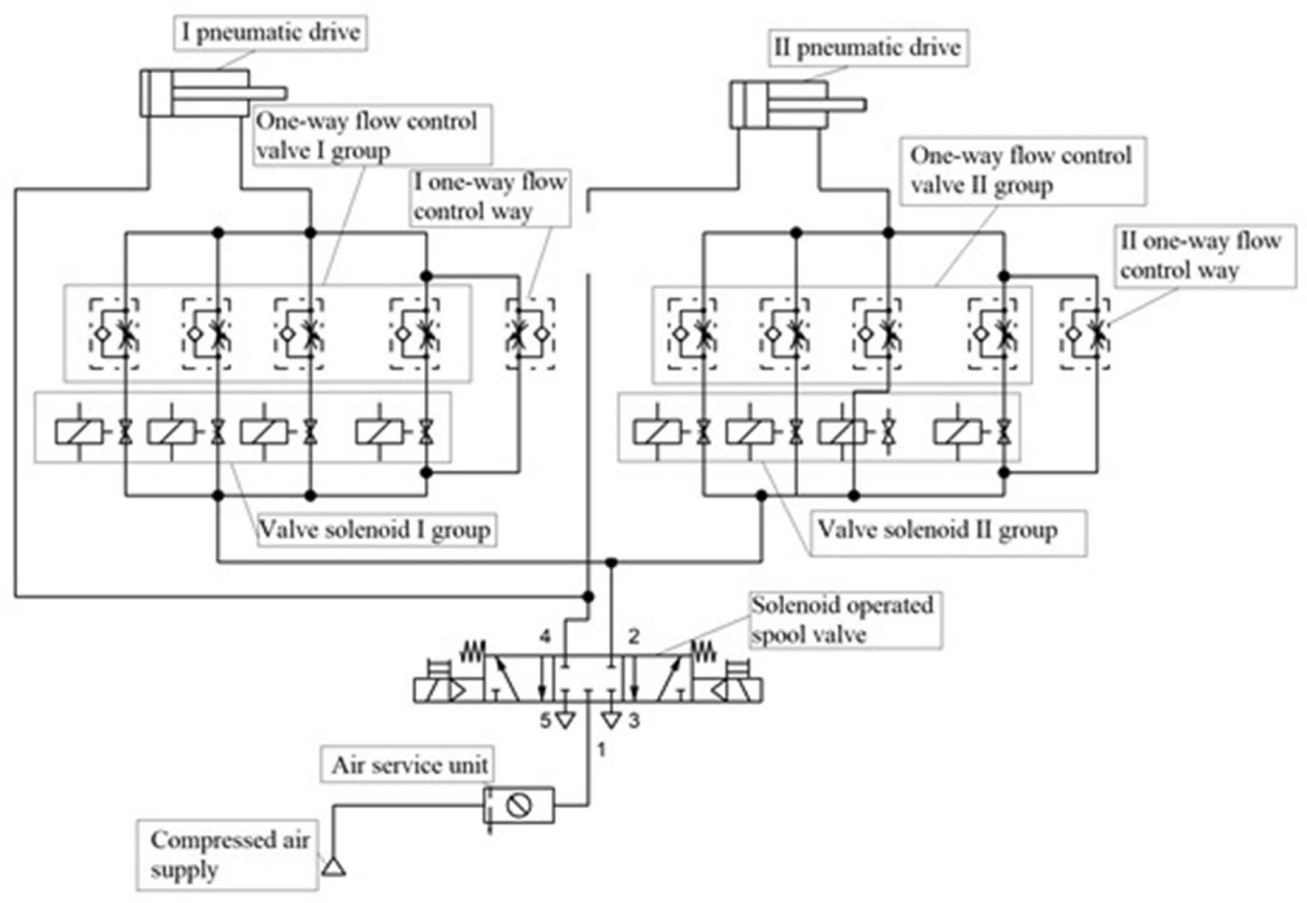
Schematic representation of the pneumatic control system used to steer the author’s patented rehabilitation device [FluidSIM-P 5.0, Festo: https://www.festo-didactic.com/int-en/services/printed-media/manuals/fluidsim-5-user-guide.htm?fbid=aW50LmVuLjU1Ny4xNy4zMi44MjguNzc1OQ].

The electronic control^29^, for which the patent was granted, is the system for controlling the extension speed of the pneumatic cylinder pistons. The diagram of the pneumatic system is shown in Fig. 1.

An important issue of the control system shown in Fig. 1 are two groups of throttle-check valves. Both groups consist of four identical throttle check valves. Throttle-check valves are properly calibrated—set at different valve opening heights.

During the extension of the actuator piston rods, the microcontroller uses potentiometric position sensors to read the difference in displacement between the two piston rods.

The microcontroller reads the difference (zone) in the displacement of the piston rods of the actuators. Then it controls the two-point valves that are connected with graduated throttle check valves and properly engaged.

**Figure 2:**
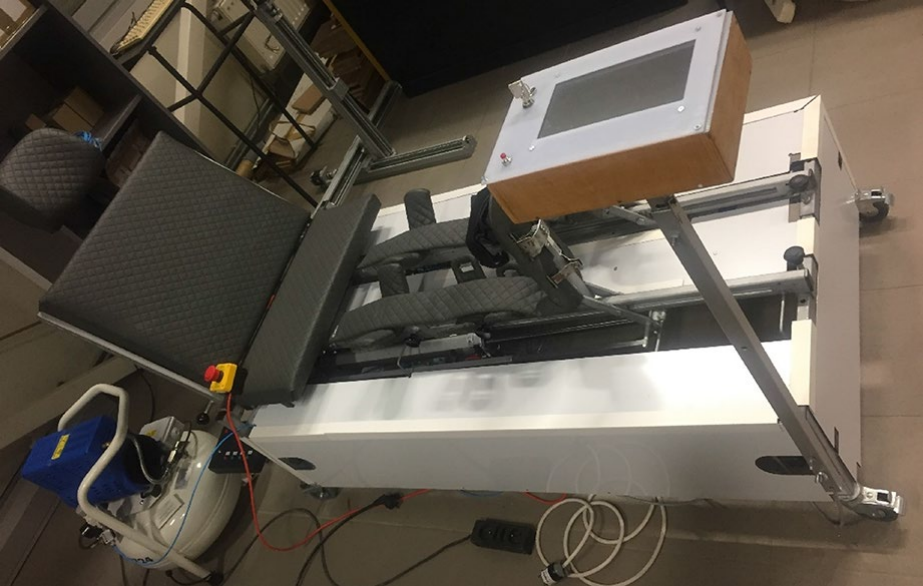
Rehabilitation device.

The electronic control^29^, can be used to regulate the concurrent movement of two cylinders in rehabilitation devices. The rehabilitation device for passive leg exercises is shown in the Fig. 2.

The patented rehabilitation device^30^ (Fig. 2) is designed to restore proper mobility of patients, e.g. after long-term immobilization following Covid-19 disease. Severely ill patients with Covid-19 spend a long period of time in hospital^31,32^. Consequently, there is a demand for such a rehabilitation device. Exercise after COVID-19 disease is very important. Exercise helps prevents muscle wasting, joint stiffness or venous thrombosis^33^ and other side effects^34–36^.

## Scaling of the throttle-check valve

In order to determine the value of the air flow through the different opening height of the throttle-check valve, the valves were calibrated. By connecting the valve to the flow meter, selecting the appropriate valve opening heights, the air flow rate was read.

### Research methodology

**Figure 3:**
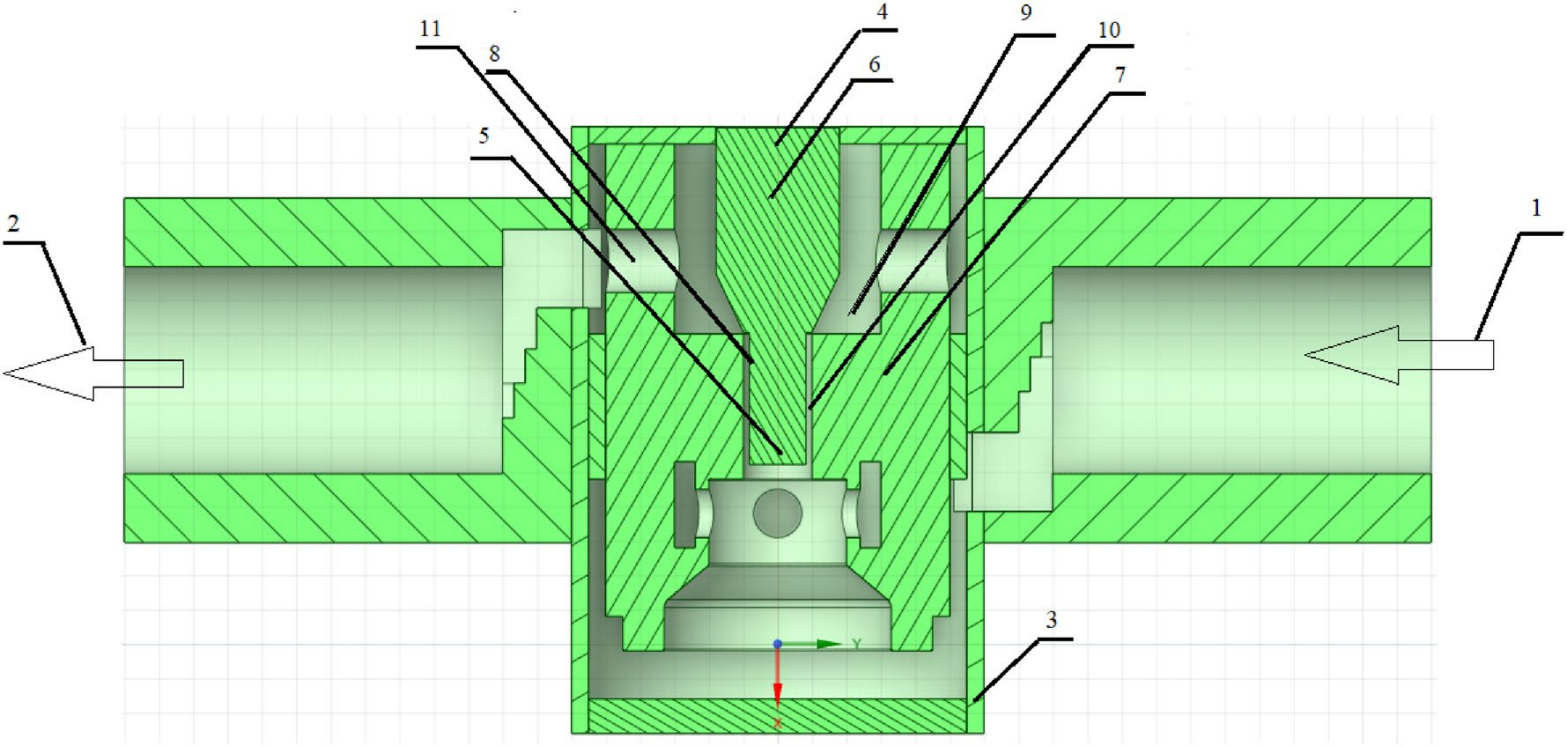
Cross section of the throttle check valve with the most important components. Where: 1—inlet channel, 2—drainage channel, 3—valve body, 4—control needle, 5—lower part of the control needle, 6—upper part of the control needle, 7—internal valve body, 8—external part of the control needle, 9—valve chamber, 10—channel choke, 11—drainage chamber [Ansys Fluent 2021 R2 (ANSYS Academic Research Mechanical and CFD): https://download.ansys.com/Current%20Release?releaseno=2021%20R2&operatingsystem=Windows%20x64].

The aim of the research was to measure the air flow rate for the appropriate opening height of the throttle valve to ensure proper operation of the pneumatic system of two pneumatic actuators. Figure 3 shows a cross-sectional view of the throttle check valve with description of key components.

In the throttle valve (Fig. 3), by changing the cross-section (changing the air flow resistance), we can control its size. This means reducing the air flow rate regardless of its direction. The amount of throttling in throttling valves with adjustable resistance is changed by the needles (4).

**Figure 4:**
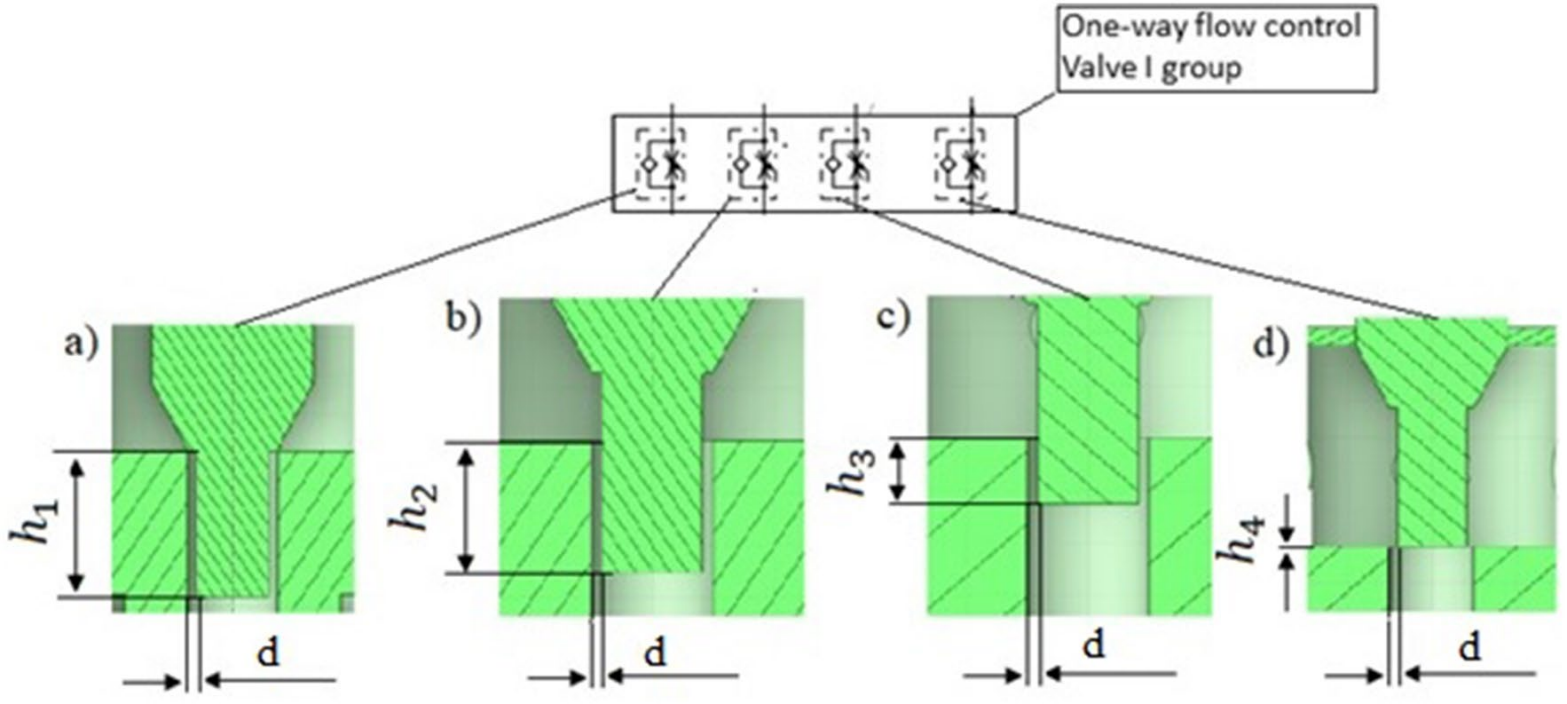
Cross sections of four opening valve heights for $d=\mathrm{0,175}\times {10}^{-3}$ m: (**a**) ${h}_{1}=3.95\times {10}^{-3}$ m, (**b**) ${h}_{2}=2.69\times {10}^{-3}$ m, (**c**) ${h}_{3}=1.43\times {10}^{-3}$ m, (**d**) ${h}_{4}=0$. [Ansys Fluent 2021 R2 (ANSYS Academic Research Mechanical and CFD): https://download.ansys.com/Current%20Release?releaseno=2021%20R2&operatingsystem=Windows%20x64].

Figure 4 below shows the cross sections of four valve heights (a) ${h}_{1}=3.95\times {10}^{-3}$ m, (b) ${h}_{2}=2.69\times {10}^{-3}$ m, (c) ${h}_{3}=1.43\times {10}^{-3}$ m, (d) ${h}_{4}=0$.

The throttle valve opening height was changed by means of the needle (Fig. 4). The valve opening heights have been set to the following values: (a) ${h}_{1}=3.95\times {10}^{-3}$ m, (b) ${h}_{2}=2.69\times {10}^{-3}$ m, (c) ${h}_{3}=1.43\times {10}^{-3}$ m, (d) ${h}_{4}=0$.

**Figure 5:**
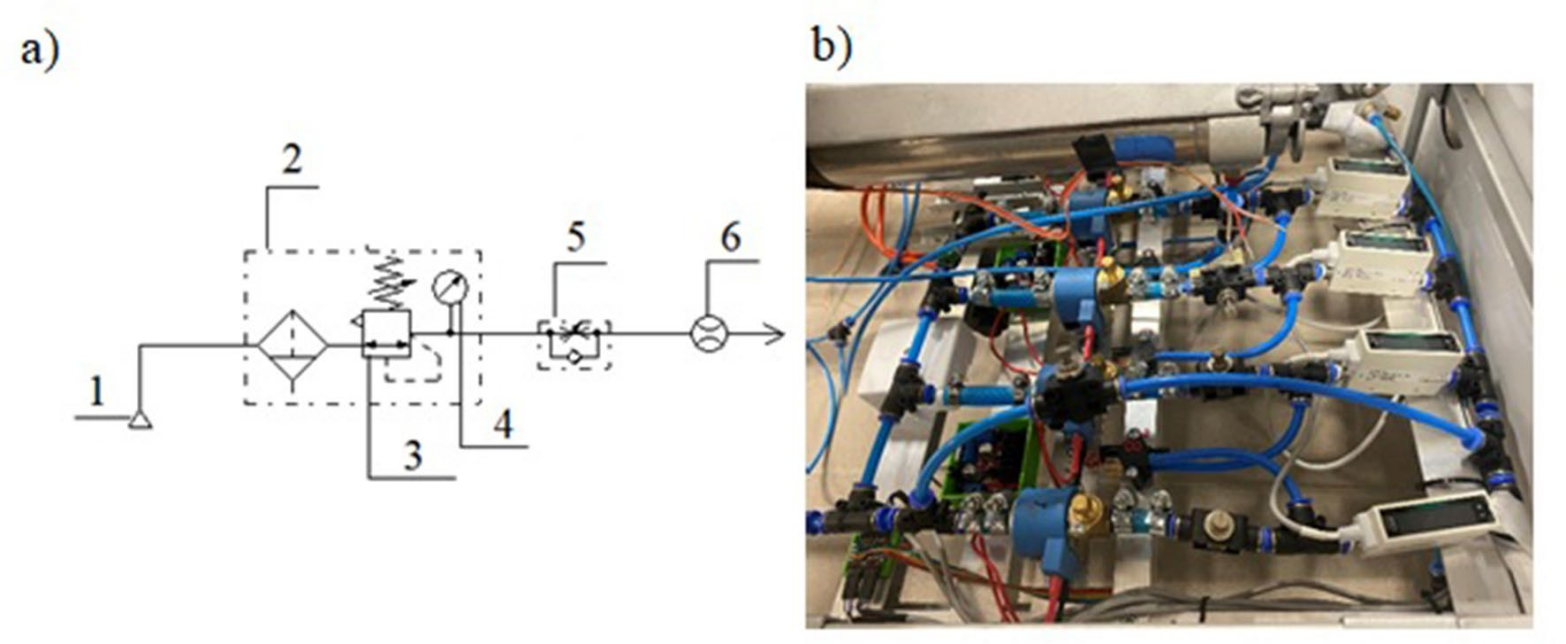
(**a**) Diagram of the test stand with a throttle-check valve: 1—power source—compressed air, 2—air preparation unit, 3—pressure reducing valve, 4—pressure gauge, 5—test element (throttle-check valve), 6—flow sensor; (**b**) The test rig. [The drawing (**a**) was created in the program: FluidSIM-P 5.0, Festo: https://www.festo-didactic.com/int-en/services/printed-media/manuals/fluidsim-5-user-guide.htm?fbid=aW50LmVuLjU1Ny4xNy4zMi44MjguNzc1OQ].

Figure 5a shows the test stand for testing the values air flow through the different opening height of the throttle-check valve. Figure 5b shows the test rig.

A flow sensor 6 was installed on the pneumatic lines (Fig. 5a above) leading to the throttle-check valve. The sensor is used to read the values of the air flow rate. The measuring stand was supplied with compressed air 1, supplied by the air preparation unit 2, the pressure of which was regulated by the reducing valve 3. The pressure gauge 4 serves as the indicator of the system pressure values.

**Figure 6:**
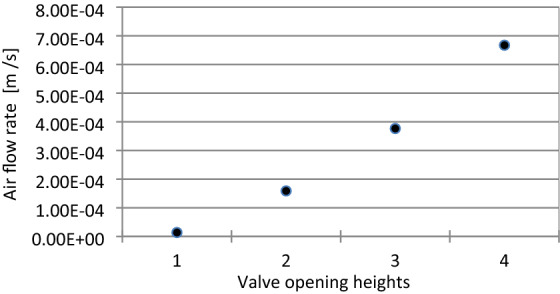
Relation between throttle opening values and different valve opening heights. Where: 1$-{h}_{1}=3.95\times {10}^{-3}$m, 2$-{h}_{2}=2.69\times {10}^{-3}$m, 3$-{h}_{3}=1.43\times {10}^{-3}$m, 4 $-$ ${h}_{4}=0$.

Figure 6 shows a diagram illustrating the relationship between the air flow rate and the opening height of the throttle valve. Readings were made for the supply pressure value of $3.5\times {10}^{5}$ Pa.

At a supply pressure (Fig. 6) of $3.5\times {10}^{5}$ Pa and the first opening height of the throttle valve ($3.95\times {10}^{-3}$ m), the value of the air flow rate is $1.36\times {10}^{-5}{\mathrm{m}}^{3}/\mathrm{s}$, while at the second throttle valve opening height ($2.69\times {10}^{-3}$ m) the value of the air flow rate is $1.59\times {10}^{-4} {\mathrm{m}}^{3}/\mathrm{s}$. For the third throttle valve opening height ($1.43\times {10}^{-3}$ m) the value of the air flow rate is $3.77\times {10}^{-4}{\mathrm{m}}^{3}/\mathrm{s}$, and for the fourth throttle valve opening height ($0$ m) the value of the air flow rate is $6.67\times {10}^{-4} {\mathrm{m}}^{3}/\mathrm{s}$.

## Mathematical models

Any physical process involving fluid flow can be described by a mathematical model. For this purpose, the Navier Stokes equations are used, which include: mass conservation Eq. (1), energy Eq. (2) and momentum conservation Eq. (3). When conducting numerical simulations, an important factor is the selection of an appropriate model that will be as close as possible to real phenomena^37^.

The air (ideal gas) flowing through the analyzed pneumatic valve is treated as a continuous medium. Additionally, it has the features of a Newtonian fluid.

Mass conservation equation:

1 $$\frac{\partial \rho }{\partial t}+\frac{\partial }{\partial {x}_{j}}\left(\rho {u}_{j}\right)=0$$

Energy equation:

2 $$\frac{\partial e}{\partial t}+\frac{\partial }{\partial {x}_{j}}\left({u}_{j}\left(e+p\right)-{u}_{i}{\tau }_{ij}-{q}_{j}\right)=0$$

Momentum conservation equation:

3 $$\frac{\partial \rho {u}_{i}}{\partial t}+\frac{\partial }{\partial {x}_{j}}\left({\rho u}_{j}{u}_{i}+p{\delta }_{ij}-{\tau }_{ij}\right)=0$$

Ideal gas law equation:

4 $$p=\rho RT$$

where: e—specific energy, p—static pressure, R—universal gas constant, t—time, T—temperature, ${u}_{i,j}$—mean flow velocity component in the ${x}_{i,j}$ direction, ${x}_{i,j}$—coordinate component, ${\delta }_{ij}$—2nd order Kronecker tensor, $\rho$—density, ${\tau }_{ij}$—stress tensor.

### Turbulence model

The phenomenon of turbulence is a key issue in fluid dynamics, and the accuracy of the models used is reflected in the correctness of simulation of complex turbulent flows. Turbulence models developed by scientists are validated against experimental data to test their performance under various flow conditions^37,38^. The k-ω SST model is similar to the standard k-ω model.

Transport equation for turbulent kinetic energy *k* is as follows:

5 $$\frac{\partial \rho k}{\partial t}+\frac{\partial }{\partial {x}_{j}}\left(\rho k{u}_{j}-(\mu +{\sigma }_{k}{\mu }_{t}\right)\frac{\partial k}{\partial {x}_{j}})={\tau }_{tij}{S}_{ij}-{\beta }^{*}\rho \omega k$$

Transport equation for specific dissipation of kinetic energy of turbulence $\omega$:

6 $$\frac{\partial \rho \omega }{\partial t}+\frac{\partial }{\partial {x}_{j}}\left(\rho \omega {u}_{j}-(\mu +{\sigma }_{\omega }{\mu }_{t}\right)\frac{\partial \omega }{\partial {x}_{j}})={P}_{\omega }-\beta \rho {\omega }^{2}+2(1-{F}_{1})\frac{\rho {\sigma }_{\omega 2}}{\omega }\frac{\partial k}{\partial {x}_{j}}\frac{\partial \omega }{\partial {x}_{j}}$$

Absolute value of vorticity appearing in equation (6), is defined as follows:

7 $${P}_{\omega }\equiv 2\gamma \rho \left({S}_{ij}-\frac{\omega {S}_{nn}{\delta }_{ij}}{3}\right){S}_{ij}\approx \gamma \rho {\Omega }^{2}$$

Mixed function F_1_:

8 $${F}_{1}=tanh\left\{{\left(min\left[max\left[\frac{\sqrt{k}}{0.09{\omega }_{y}};\frac{500\mu }{\rho {y}^{2}\omega }\right];\frac{4\rho {\sigma }_{\omega 2}k}{C{D}_{k\omega }{y}^{2}}\right]\right)}^{4}\right\}$$

Auxiliary function F_2_:

9 $${F}_{2}=tanh\left\{{\left(max\left[2\frac{\sqrt{k}}{0.09\omega y};\frac{500\mu }{\rho {y}^{2}\omega }\right]\right)}^{2}\right\}$$

Cross-diffusion in k-ω model:

10 $$C{D}_{k\omega }=max\left[\frac{2\rho {\sigma }_{\omega 2}}{\omega }\frac{\partial k}{\partial {x}_{j}}\frac{\partial \omega }{\partial {x}_{j}};{10}^{-20}\right]$$

Turbulent viscosity is defined by the following relation as a function of turbulent kinetic energy and unit dissipation rate:

11 $${\mu }_{t}=\frac{\rho k/\omega }{max\left[1;\Omega {F}_{2}/\left({a}_{1}\omega \right)\right]}$$

where: ${a}_{1}$—constant, k—turbulent kinetic energy, ${S}_{ij}$—mean deflection rate tensor, $y$—distance from the nearest non-slip surface, $\gamma$—Poisson adiabate ratio, ${\mu }_{t}$—turbulence viscosity, ${\sigma }_{k}$,${\sigma }_{\omega }$—turbulent Prandtl numbers for k and $\omega$, $\omega$—specific turbulent dissipation rate, $\Omega$—mean vorticity value.

## Test method

The purpose of the flow analysis in the throttle-check valve was to determine the value of the fluid flow through the different opening heights of the throttle-check valve. The working medium (fluid) flow simulations were carried out for four different throttle valve slots heights.

The simulations performed will allow to obtain information on the phenomena occurring during the flow of air through various heights of the throttle valve.

### CFD analysis

Ansys (Fluent) is a common software for fluid flow simulation^39,40^. Numerical fluid mechanics allows for modeling and analysis of complex flow problems, thus enabling a better understanding of the analyzed phenomena, and optimization of existing design solutions, including pneumatic valves.

The authors of the paper^41^ used Fluent software to study internal flows in a slide-valve-type HPSV. In the article^42^, an energy-efficient high-pressure electropneumatic servo valve is presented, where a min. was carried out in CFD. flow field analysis. Computational fluid dynamics (CFD) simulations are used by the authors of the paper^43,44^ to study flow in control valves and to study the characteristics of air valves^45,46^.

Numerical simulations of the medium flow through pneumatic valves are widely used in order to evaluate the concept and optimize their operation, as well as to understand the physical phenomena occurring in individual design solutions. They make it possible to obtain information not only about the local values of relevant physical quantities, such as e.g. temperature or pressure, but also to determine the distribution of thermodynamic parameters in the considered volume (fluid domain). Due to the small size and construction of some valves, the use of available measurement methods is sometimes troublesome or requires the use of advanced measurement techniques. Therefore, the use of numerical fluid dynamics methods is justified in this case.

Numerical calculations using CFD (Computational Fluid Dynamics) methods are currently a dynamically developing computational technique. These tests are very helpful at the stage of initial design or optimization, and contribute to the reduction of the amount and costs of experimental research.

The use of numerical fluid mechanics methods allowed to study the flow phenomena inside a pneumatic throttle-check valve at different sizes of flow gaps.

**Figure 7:**
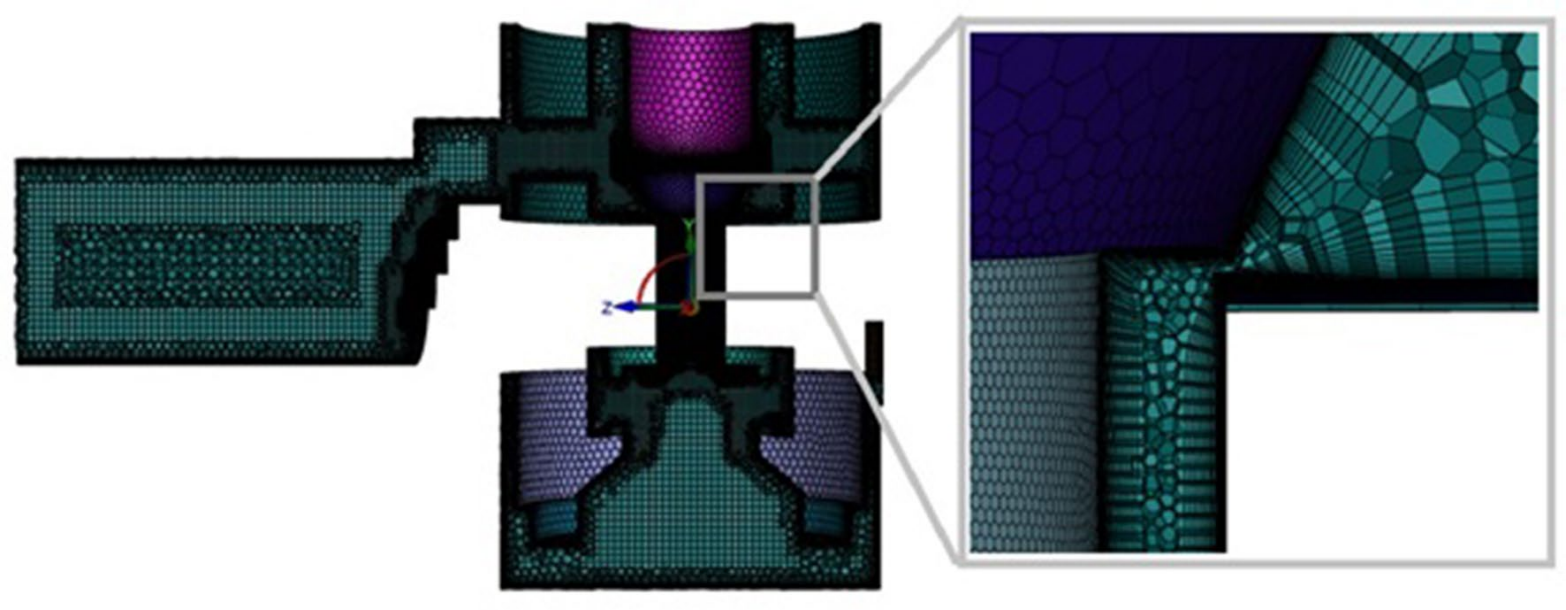
Computational grid—poly-hedral elements for case number 1. [Ansys Fluent 2021 R2 (ANSYS Academic Research Mechanical and CFD): https://download.ansys.com/Current%20Release?releaseno=2021%20R2&operatingsystem=Windows%20x64].

For the purpose of computer simulations, the simplified three-dimensional geometry of the throttle valve was prepared in the Ansys SpaceClaim environment, based on the actual geometric dimensions. The separated computational area was discretized using poly-hexcore elements in Fluent Meshing (Fig. 7). The use of multi-walled elements used in Mosaic technology ensures, a high-quality transition between meshes of various types.

The comparative analyzes carried out show^47^ that the use of Mosaic technology allows to reduce the size of the computational mesh, which, combined with the increase in mesh quality, contributes to the reduction of the computation time and enables a better solution accuracy^47^. In the areas where large gradients occur, the finite volume mesh was locally compacted using the gradient mesh adaptation technique, where the fundamental parameter was the velocity gradient of the flowing liquid.

Detailed information on the mesh size for the analyzed cases is provided in Table 1.

**Table 1:** Mesh size.

	Case 1	Case 2	Case 3	Case 4
Valve opening heights (m)	3.95 × 10^–3^	2.69 × 10^–3^	1.43 × 10^–3^	0
Number of cells	3,105,134	3,815,832	5,059,893	5,683,578
Number of boundary layers	15	15	20	20

The next step was to define the boundary conditions and determine the parameters of the fluid flowing through the valve. Then the conditions for conducting numerical analysis were determined.

The analysis of the medium flow through the valve was performed with the use of Ansys Fluent software. The purpose of the analysis is to determine the flow characteristics. The tests were carried out for various values of the mass flow rate and for different heights of the throttle valve opening.

The independence test on the mesh grids was carried out to find the right size^48^. The results are shown in Table 2 for case number 1, when the gap height is $3.95\times {10}^{-3}$ m. Analyses were carried out in a similar manner for the other cases considered. Three mesh sizes were tested to determine the effect on the velocity of the medium flowing through the valve.

**Table 2:** Grid independence study.

	Number of cells	Velocity (m/s)
1	2,320,134	64.78
2	3,105,134	66.02
3	3,731,577	67.09

Based on the results obtained, it was concluded that the solution is independent of the mesh size. Therefore, for the numerical simulation of the first case analyzed, a grid size of 3,105,134.

### Boundary conditions

Defining the boundary conditions that will allow to correctly describe the spectrum of flow phenomena occurring in the analyzed throttle valve is an essential stage of the simulation research. It was assumed that the analyzed phenomena are steady state conditions. Pressure-based solver type was selected. Boundary conditions were determined on the basis of experimental tests. The value of the supply pressure was assumed to be 350 kPa. The operating pressure was set at 101.325 Pa.

The values of the mass flow rate for individual valve opening heights are presented in Table 3.

**Table 3:** Mass flow rate.

	The height of the slot	Mass flow inlet
Analyzed case	$\times {10}^{-3}$ (m)	$\times {10}^{-5}$ (kg/s)
1	$3.95$	1.667
2	$2.69$	19.452
3	$1.43$	46.129
4	0	81.729

A three-dimensional computational domain was used in the simulation. The working medium, the air, is described using the ideal gas model. A constant value of 300 K for the temperature on the walls and constant viscosity of the fluid were assumed. The conducted numerical analysis does not take into account the phenomena related to heat transfer. A mass flow inlet boundary condition was assigned to the inlet, and a pressure outlet condition was assigned to the outlet. The k − ω SST shear stress transport model, developed by Menter^37^, was used. This model combines the advantages of commonly used models such as the k − ε Two-Equation Model proposed by Launder-Sharm and the k − ω Two-Equation Model proposed by Wilcox^37^ and can be used to model phenomena related to internal fluid flow^49,50^. No-slip conditions were assumed on all walls of the valve.

The k − ω SST model used is now a common turbulence model used in numerical analysis. It is based on the k-ω Standard model and the k − ε model. The k − ω model is well suited to simulate flow in the viscous sub-layer, while the k − ε model gives a better representation of flow behavior in regions away from the wall. These features make it more accurate for a wider range of flows. Therefore, in order to model the internal flow through the throttling-return valve, it was decided to choose the k − ω SST model because it allows more accurate modeling of the phenomena both inside the near-wall layer as well as outside the near-wall layer in the free-flow region of the fluid^51,52^.

The obtained results allowed to determine the distribution of relevant physical quantities such as static pressure, velocity of the medium flowing through the valve or vector velocity distribution. The results are shown on the XY plane, coinciding with the longitudinal axis of the valve.

### Analysis of the obtained results

**Figure 8:**
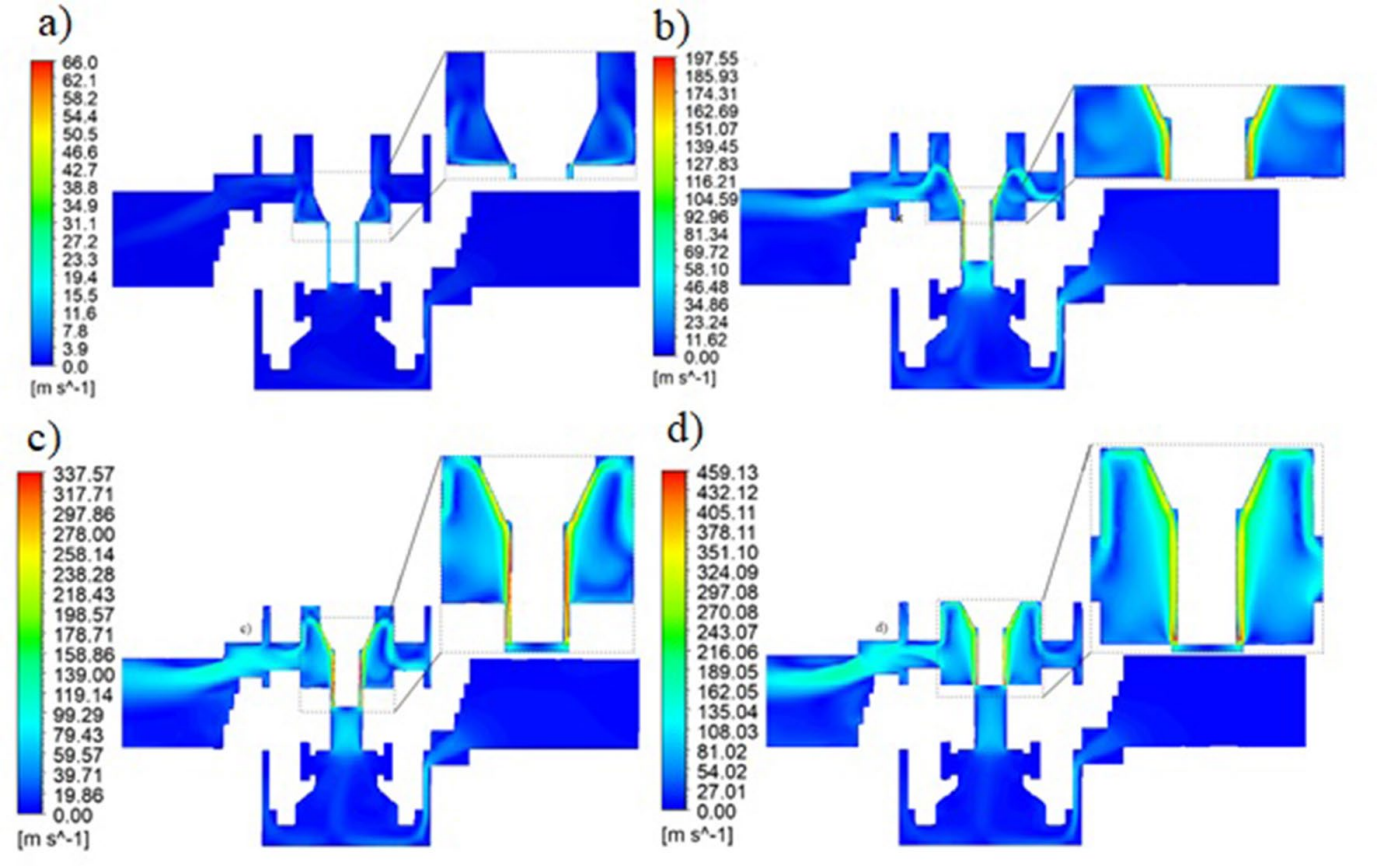
Velocity distribution for different configurations of the valve opening height; (**a**) ${h}_{1}=3.95\times {10}^{-3}$ m, (**b**) ${h}_{2}=2.69\times {10}^{-3}$ m, (**c**) ${h}_{3}=1.43\times {10}^{-3}$ m, (**d**) ${h}_{4}=0$ [Ansys Fluent 2021 R2 (ANSYS Academic Research Mechanical and CFD): https://download.ansys.com/Current%20Release?releaseno=2021%20R2&operatingsystem=Windows%20x64].

Numerical simulation (CFD) makes it possible to study the flow phenomena inside a pneumatic throttle-check valve, with different sizes of flow gaps. The following valve opening heights were modeled: (a) ${h}_{1}=3.95\times {10}^{-3}$ m, (b) ${h}_{2}=2.69\times {10}^{-3}$ m, (c) ${h}_{3}=1.43\times {10}^{-3}$ m, (d) ${h}_{4}=0$. Figure 8 shows the velocity distribution for different valve opening heights.

As the cross-sectional area decreases, the velocity of the flowing fluid increases. Based on the analysis of the velocity distribution (Fig. 8), it can be seen that the highest velocities are obtained in the choke channels between the needle and the internal valve body.

**Figure 9:**
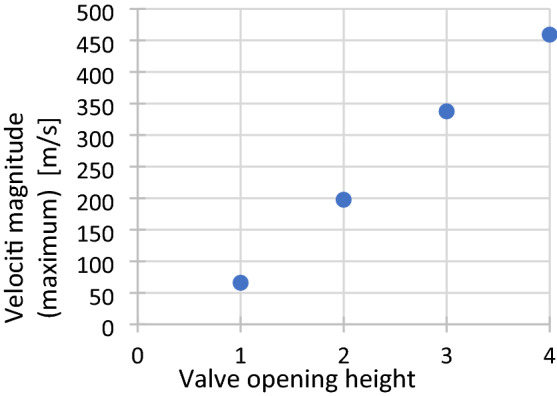
Maximum speed for various configurations of the valve opening height for: Where: 1$-{h}_{1}=3.95\times {10}^{-3}$m, 2$-{h}_{2}=2.69\times {10}^{-3}$m, 3$-{h}_{3}=1.43\times {10}^{-3}$m, 4 $-$ ${h}_{4}=0$ m.

Figure 9 shows the maximum air flow velocities obtained in the area with the smallest cross-sectional area (in the throttle channel), for all four considered cases. In the analyzed problem, a linear increase in the speed in the gap can be noticed for the given operating conditions of the valve.

At the first opening height of the throttle valve (Fig. 9), the maximum speed in the gap is 66.0 m/s. At the second throttle valve opening height, the value of the maximum air flow velocity is 197.55 m/s. For the third throttle valve opening height, the value of the maximum speed is 337.57 m/s. At the fourth throttle valve opening height, the maximum flow velocity is 459.13 m/s.

**Figure 10:**
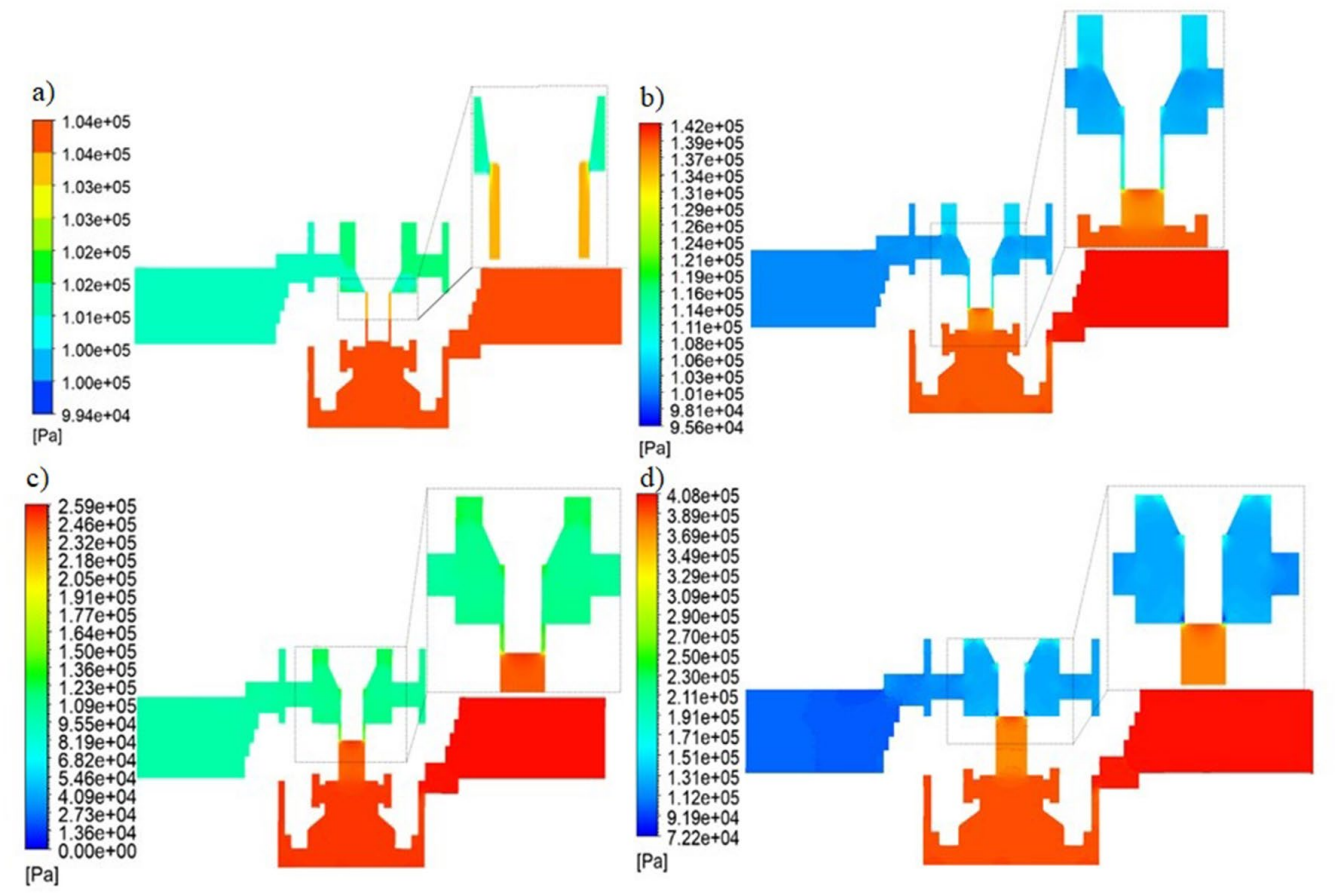
Static pressure for various configurations of the valve opening height. (**a**) ${h}_{1}=3.95\times {10}^{-3}$ m, (**b**) ${h}_{2}=2.69\times {10}^{-3}$ m, (**c**) ${h}_{3}=1.43\times {10}^{-3}$ m, (**d**) ${h}_{4}=0$ [Ansys Fluent 2021 R2 (ANSYS Academic Research Mechanical and CFD): https://download.ansys.com/Current%20Release?releaseno=2021%20R2&operatingsystem=Windows%20x64].

Figure 10 shows changes in static pressure occurring in the throttle valve for different valve opening heights.

Static pressure allows for the analysis of pressure losses in the tested object. Analyzing the four cases of valve geometry, it can be seen that the maximum value of static pressure occurs in the inlet channel of the valve. A sudden drop in static pressure is noticeable in the choke channel between the needle and the internal valve body. The decrease in static pressure is accompanied by an increase in the velocity of the air flowing through the gap (Fig. 10), which is consistent with the Bernoulli equation.

Table 4 summarizes the averaged values of the static pressure at the inlet and at the outlet of the analyzed valve, and the static pressure losses that occur in the flow between the outlet and the inlet to the valve.

**Table 4:** Static pressure losses in the considered flow field.

	The height of the slot	Static pressure (inlet)	Static pressure (outlet)	delta p
Case	$\times {10}^{-3}$ (m)	Pa	Pa	Pa
1	$3.95$	104,197.82	101,325.00	2,872.82
2	$2.69$	141,968.38	101,322.96	40,645.42
3	$1.43$	259,196.21	101,316.70	157,879.51
4	0	408,261.54	101,302.67	306,958.87

Analyzing the obtained results (Table 4), it can be noticed that with the change in the size of the throttling channel, which is related to the increase in the mass flow rate, the pressure loss value increases.

**Figure 11:**
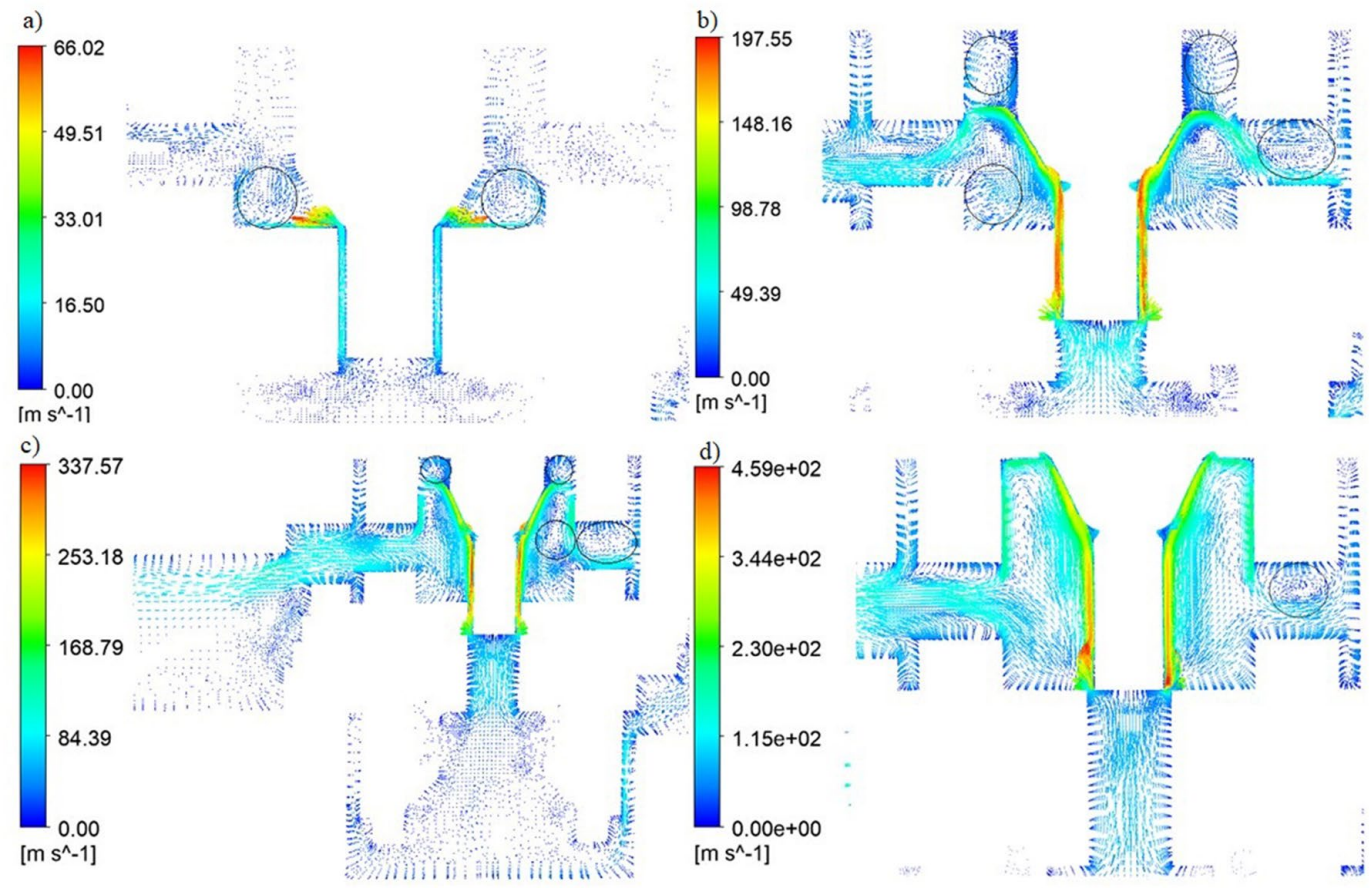
Velocity vectors for slots with a height of (**a**) ${h}_{1}=3.95\times {10}^{-3}$ m, (**b**) ${h}_{2}=2.69\times {10}^{-3}$ m, (**c**) ${h}_{3}=1.43\times {10}^{-3}$ m, (**d**) ${h}_{4}=0$. [Ansys Fluent 2021 R2 (ANSYS Academic Research Mechanical and CFD): https://download.ansys.com/Current%20Release?releaseno=2021%20R2&operatingsystem=Windows%20x64].

Figure 11 shows the vector velocity distribution for the four analyzed valve opening ranges. The produced vortices are marked with circles in the figures.

Velocity vectors (Fig. 11a) allow to indicate areas characterized by a pressure drop caused by disturbances in the flow. When analyzing the obtained results, it can be noticed that there are zones in the flow field with local turbulences of the working medium flowing through the valve. You can also observe areas where the boundary layer is detached. The occurrence of disturbances in the flow contributes to the increase in pressure losses. The formation of disturbances is influenced by the internal geometry of the valve—as a result, it changes the speed and direction of air flow (e.g. sharp edges).

For the second case of opening the valve (Fig. 11b), asymmetrical flow disturbances were noticed. One can observe vortices formed both in the lower part of the valve chamber and in the upper part of the valve chamber and in the inflow chamber.

While analyzing Fig. 11c as in the previous case, vortices in the outflow chamber were noticed. Additionally, they are also present in the upper part of the valve chamber and at the bottom of the valve chamber.

Visible disturbances (Fig. 11d) in the flow were also noted in the fourth case under examination. Eddies were observed only in the drainage channel.

When analyzing all the considered cases (Fig. 11a–d), it can be noticed that the smallest flow disturbances occur in the fourth case. On the other hand, the greatest disturbances occur for the second valve opening height. Analysis of the flow results for the valve shows that the high velocity fields in all four cases are located between the outside of the needle and the throttle channel.

**Figure 12:**
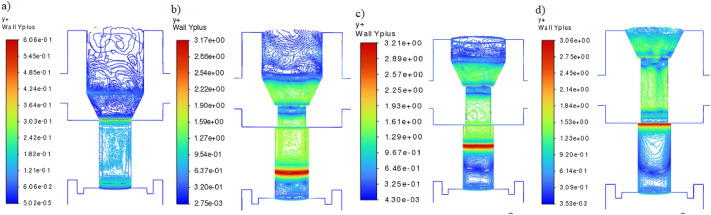
Y + for the following cases: (**a**) following cases: (**a**) ${h}_{1}=3.95\times {10}^{-3}$ m, (**b**) ${h}_{2}=2.69\times {10}^{-3}$ m, (**c**) ${h}_{3}=1.43\times {10}^{-3}$ m, (**d**) ${h}_{4}=0$. [Ansys Fluent 2021 R2 (ANSYS Academic Research Mechanical and CFD): https://download.ansys.com/Current%20Release?releaseno=2021%20R2&operatingsystem=Windows%20x64].

Figure 12 shows the y + parameter for the four cases considered.

The y + parameter (wall function y +), read for the most important area of the throttle valve (on the outer surface of the control needle), did not exceed the value of 5. In the remaining areas, the y + parameter was less than 1^50^.

### Summary of analyses

- The obtained flow analysis results are of fundamental importance for the operation of the group of valves located in the motion control system of the two pneumatic actuators in a rehabilitation facility.
- Fig. 9 shows the maximum velocity values of the air flow through the valve, obtained in the smallest section. Based on the results of numerical analysis, it can be concluded that the airflow reaches the transonic velocity for the opening of the valve to the height of ${h}_{3}=1.43\times {10}^{-3}$ m and supersonic value when opening the valve to height ${h}_{4}=0$ m. This causes the airflow in this area to be stifled. In a throttled flow, the airflow velocity will not increase in the constriction (the area with the smallest flow area), while the pressure drop between the inlet and outlet of the valve is significant. For the other cases analysed, when opening the valve to a height ${h}_{1}=3.95\times {10}^{-3}$ m and ${h}_{2}=2.69\times {10}^{-3}$ m the air flowing through the valve reached subsonic velocities. It is recommended that the valve opening height for the fourth case be changed to avoid airflow at supersonic speeds.
- Flow analyses influence the understanding of the interaction between the flow of medium–air (compressible medium) and the geometry of the valve.
- Flow analysis not only explains the underlying mechanisms of valve flow dynamics, but also provides important guidance on changes in flow intensity at different valve openings.
- Numerical tests will allow for appropriate scaling of the group of throttling valves in the control system of the movement of two drives in the rehabilitation device, which will be the subject of further studies.

## Conclusions

An important issue is the use of throttle valves in the control system of pneumatic drives for rehabilitation devices. The control system for the concurrent movement of two actuators presented in the article is used in a rehabilitation device for passive exercises of the lower limbs. Such a device is intended for patients with locomotor dysfunctions, mainly for the rehabilitation of the knee and hip joints. Simultaneous movement of both lower limbs in passive and active exercises is aimed at, among others, developing and maintaining a full range of motion in the joints, preventing the formation of muscle contractions and preventing pressure ulcers. This device is also very useful for the rehabilitation of patients who have developed a severe course of disease caused by the Covid-19 virus.

The rehabilitation device simulates the natural movement of the limbs by means of actuators. The control system used in the device allows the simultaneous movement of two piston rods of pneumatic actuators, and the innovative control regulates the concurrent movement of two actuators.

Therefore, it is important to select a rehabilitation device with properly calibrated throttle valves for the control system.

The simulation carried out in the Ansys Fluent software allowed to study the flow phenomena at different sizes of the throttle-check valve gaps. The velocity and pressure fields as well as the vector velocity distribution of the flowing fluid were determined. The obtained results will allow for a possible modification of the throttle-check valve geometry so as to obtain a more favorable distribution of physical quantities at different heights of the flow gaps. The greatest drop in static pressure was observed in the area behind the throttle valve slot, which therefore leads to an increase in the velocity of the air flowing through the throttle channel. This situation applies to all analyzed cases. Both the mass flow rate and the pressure loss value increase alongside with the throttle channel size.

The performed numerical tests constitute the basis for further research aimed at determining the optimal geometry of the pneumatic valve, using the Fluent Adjoint Solver method, which aims to reduce pressure losses in the flow.

## Data Availability

The datasets used and/or analyzed during the current study available from the corresponding author on reason-able request.
